# Dispositional empathy predicts primary somatosensory cortex activity while receiving touch by a hand

**DOI:** 10.1038/s41598-021-90344-x

**Published:** 2021-05-28

**Authors:** Michael Schaefer, Anja Kühnel, Franziska Rumpel, Matti Gärtner

**Affiliations:** 1 Medical School Berlin, Calandrellistr. 1-9, 12247 Berlin, Germany; 2 Otto-Von-Guericke Business School Magdeburg, 39106 Magdeburg, Germany; 3 Universitätsmedizin Berlin, Department of Psychiatry and Psychotherapy, Campus Benjamin Franklin, Hindenburgdamm 30, 12203 Berlin, Germany

**Keywords:** Psychology, Human behaviour

## Abstract

Previous research revealed an active network of brain areas such as insula and anterior cingulate cortex when witnessing somebody else in pain and feeling empathy. But numerous studies also suggested a role of the somatosensory cortices for state and trait empathy. While recent studies highlight the role of the observer’s primary somatosensory cortex when seeing painful or nonpainful touch, the interaction of somatosensory cortex activity with empathy when receiving touch on the own body is unknown. The current study examines the relationship of touch related somatosensory cortex activity with dispositional empathy by employing an fMRI approach. Participants were touched on the palm of the hand either by the hand of an experimenter or by a rubber hand. We found that the BOLD responses in the primary somatosensory cortex were associated with empathy personality traits personal distress and perspective taking. This relationship was observed when participants were touched both with the experimenter’s real hand or a rubber hand. What is the reason for this link between touch perception and trait empathy? We argue that more empathic individuals may express stronger attention both to other’s human perceptions as well as to the own sensations. In this way, higher dispositional empathy levels might enhance tactile processing by top-down processes. We discuss possible implications of these findings.

## Introduction

The last decades have seen an ever-increasing number of studies on the conditions of empathic behavior. Nevertheless, current research still lacks a clear definition of empathy. Most researchers describe empathy as our capacity to understand the emotions and intentions of others and to relate to our conspecifics. For example, de Waal et al. proposed the perception–action model (PAM), which defines empathy as the ability to imagine a situation from the other person’s point of view and the sharing of emotions^1^. Many (but see^2^) theoretical conceptualizations of empathy agree with the assumption that empathy includes both cognitive as well as affective components, thus enabling us to vicariously experience the feelings and understand the given situation of another^3,4^.

Recent research also addressed the neural underpinnings of both state and trait empathy. Given the various experimental contexts, different neural substrates for empathy have been discussed. Many studies suggest a main network including in particular the bilateral anterior insula and the anterior/midcingulate cortex (ACC/MCC) for state empathy. For example, when participants witness somebody else in pain and feel empathy, studies showed an engagement of these brain regions. Activity in those brain regions have been related to personal distress and perceived pain intensity^5,6^. However, recent research showed that the ACC shows different activation during felt and observed pain when examining unsmoothed data, suggesting neighboring but distinct cortical activations here^7^. The ACC/MCC may reflect modulations of motor processing when seeing other’s pain, as suggested by several studies^8–12^. In contrast, the insula shows consistently activation both in felt and observed pain and touch^13–15^.

Studies have also addressed the neural correlates of empathy as a stable personality trait. For example, Banissy et al. reported differences in grey matter volume for affective empathy (empathic concern and personal distress) in the ACC and insula, and also in precuneus and somatosensory cortex. Trait differences for the cognitive component of empathy (perspective taking and fantasy) were associated with activations in the ACC and dorsolateral prefrontal cortex^16^ (similar^17–19^). While Banissy et al. and others used voxel-based morphology (VBM) to reveal trait empathy differences, a recent study examined markers of myeloarchitectural integrity and found differences in insular and somatosensory cortex for empathy personality traits^20^. Furthermore, Peled-Avron et al. used an EEG-approach to show that affective empathy (personal distress) is related to electrophysiological responses to observed social touch^21^.

The neural underpinnings of empathy have also been discussed by studies on autism spectrum disorders. Alterations in somatosensory processing are often reported in individuals with autism spectrum disorders^22,23^. Studies examining touch processing in autism showed atypical processing for affective and nonaffective touch. For example, Kaiser et al. reported enhanced responses for nonaffective touch in SI, which they linked to atypical sensory cortical hyper-reactivity^24^. In addition, Khan et al. reported abnormalities in functional connectivity of the somatosensory cortices (both SI and SII)^25^.

Both for state and for trait empathy an engagement of the somatosensory cortices (SI and SII) is suggested. What is the contribution of these brain regions to empathic feelings? Recent studies reported that our somatosensory cortices are involved when we see the injured body part, but not when we observe facial expressions or more abstract signs of someone in pain (e.g.,^5,26–29^). These findings suggest a resonating role of the somatosensory cortices. Beyond the observation of others in pain, studies also showed that viewing nonpainful touch is associated with an activation of the observer’s somatosensory cortex (e.g.,^30–33^, but see^34^). These mirror-like activations in somatosensory cortices when observing others being touched have been shown to be linked with the empathic abilities of the observer. For example, BOLD responses in SI were associated with empathy personality traits (perspective taking) (e.g.,^35,36^). However, the exact roles of the somatosensory cortices for empathy are still a matter of discussion^37^.

While the above-mentioned studies suggest a contribution of the somatosensory cortex (in particular SI) to empathy when seeing someone else in pain or simply observing someone who receives nonpainful touch, it is unknown whether receiving touch on the own body may also be linked to dispositional empathy. Why should feeling touch on the own body be related to empathy personality traits? One could speculate that more empathic individuals may not only pay more attention to other’s feelings but also to the own sensations. Recent studies on using meditation (paying attention to the own body) to improve empathy (paying attention to (the body of) others) found some support for this idea (e.g.,^38,39^). However, in contrast to this top-down view a bottom-up process might also be possible. Thus, somatosensory activation might cause higher attention and thereby result in higher empathy scores (e.g.,^28,40^).

The present study aims to exploratory examine whether there is a correlative relationship between touch-related activity in somatosensory cortex activity and empathic personality traits. We scanned participant’s brain activity while an experimenter touched the palm of the participant’s hand. In addition, the experimenter touched the participant with a rubber hand, which serves as a control condition (the participant receives similar intentional touch but does not feel a real human hand). We then examined the relationship of brain activation in somatosensory cortices with dispositional empathy and Big Five personality traits.

Given that previous studies found that age affected both empathy^41–43^ as well as tactile processing^43–45^, we added age as a further variable in our analyses.

## Materials and methods

### Participants

29 people (17 females) with a mean age of 22.17 years (± 3.32 standard deviation) took part in the study. All participants were right-handed native German volunteers and had no neurological or psychiatric history. The study adhered to the Declaration of Helsinki. An ethical approval was obtained from the ethics committee of the Medical School Berlin (Germany). All participants gave written informed consent to the study.

### Procedure

While lying in the scanner participants received touch by a real hand, touch by a rubber hand, or no touch. In the real hand touch condition an experimenter touched the palm of the participant’s right hand for about 10 s. In this time the experimenter caressed the participant’s hand several times. In the rubber hand touch condition touch was applied (by the experimenter) in a similar way, but here we used a life-sized rubber hand. This hand was made out of soft rubber material and could slightly be bended such as a real hand. This condition was motivated by applying similar intentional touch as in the real hand touch condition, but here with only minor affective components. In the no touch condition no tactile or other stimuli were presented. All three conditions were randomly assigned. After being touched subjects were asked to rate the strength of the felt touch (2 s) and how pleasant it felt to them (2 s). Participants responded with their left hand using a key with four buttons (Likert-scale from 1 to 4, strength: 1 = not at all strong, 4 = very strong; pleasantness: 1 = not at all pleasant, 4 = very pleasant). After responding there was a break of 12 s.

The experiment consisted of four runs, with each run including all three conditions (randomized). Conditions were repeated 5 times for each run. The experiment lasted about 45 min. We placed foam cushions tightly around the side of the subject’s head to minimize head motion. Visual stimuli were presented on a visual display inside the scanner using LCD glasses.

After scanning (on a separate day) we asked the participants to complete two personality questionnaires, a German version of the Interpersonal Reactivity Index (IRI)^46,47^, and a German version of the NEO Five-Factor Inventory (NEO-FFI)^48,49^.

The IRI measures self-reported empathic behavior, it is widely used and extensively validated (e.g.,^6,50^). The 28-item questionnaire consists of four subscales. The scale perspective taking (PT) represents the tendency to think from another perspective. A subscale fantasy (F) measures the participant’s ability to transpose oneself into the feelings and actions of fictional characters in books or movies. The scale empathic concern (EC) describes feelings of compassion or sympathy for others. The subscale personal distress (PD) measures the propensity to have aversive emotional feelings when witnessing distress in others. According to Davis, EC and PD can be described as the affective component, whereas PT and F measure the cognitive component of empathy^47^.

To further examine the relationship between touch related somatosensory activity and personality we asked participants to complete the NEO-FFI to assess the Big Five personality traits. This questionnaire includes 60 items and is based on a factor-analytic approach, which describes the human personality in five core dimensions: extraversion, neuroticism, agreeableness, conscientiousness, and openness to experience. The dimension neuroticism describes the experience of negative emotions including anxiety, self-consciousness, and irritability. Extraversion is displayed by a tendency to experience positive emotions and linked to sociability, assertiveness, and talkativeness. Agreeableness is linked towards a tendency to altruism, cooperation, compassion, and politeness. The dimension conscientiousness is reflected by disciplined, organized, and achievement-oriented behavior. Openness to experience describes active imagination, aesthetic sensitivity, attentiveness to inner feelings, preference for variety, and intellectual curiosity^48^.

### FMRI data acquisition and analysis

MRI data were acquired using a 3T Siemens Tim Trio scanner (Siemens, Germany). High-resolution T1-weighted structural images for anatomic reference were acquired using an MP-RAGE sequence prior the functional runs (TR = 1650 ms, TE = 5 ms). Whole brain T2-weighted functional images were then collected using gradient echo-planar images (TR = 2 s, TE = 35 ms, flip angle = 80°, FOV = 224 mm, number of slices = 32, voxel size = 3.125 × 3.125 mm). Data were acquired in four runs, each consisting out of 365 brain volumes.

Imaging data were preprocessed and analyzed using the Statistical Parametric Mapping Software (SPM12, Wellcome Department of Imaging Neuroscience, University College London, London, UK). For each subject the fMRI scans were realigned to correct for inter-scan movement, using sinc interpolation, and subsequently normalized into a standard anatomical space (MNI, Montreal Neurological Institute template), resulting in isotropic 3 mm voxels. Finally, data were smoothed with an 8 mm FWHM Gaussian kernel (full-width half maximum) (as described elsewhere, e.g.,^51^).

Statistical parametric maps were calculated using multiple regressions with the hemodynamic response function modeled in SPM. Data analyses were performed at two levels: First, we examined data on the individual subject level (fixed-effects-model). Second, the resulting parameter estimates for each regressor at each voxel went into a second-level analysis (random effects model) (e.g.,^51^).

We examined brain responses while participants received touch by calculating statistical comparisons for the contrast receiving touch with the real hand relative to the no touch condition and for receiving touch by the rubber hand relative to the no touch condition. We report regions that survived correction for multiple comparisons over the whole brain (at *p* < 0.05, family-wise (FWE) correction). Anatomical interpretation of the functional imaging results was performed by using the SPM anatomy toolbox.

In order to test our hypothesis we examined if there are linear relationships between personality traits (IRI dimensions, Big Five) and peak activations in SI, bilateral SII, and insula. Scores of the personality traits and age went into standard multiple linear regression analyses (separate regression models for IRI and NEO-FFI). All four IRI dimensions (or five NEO-FFI dimensions, respectively) went simultaneously into the model. We used the software package SPSS for calculating regression analyses (IBM Corp., Armonk, NY, USA).

## Results

### Behavioral results

Table 1 displays mean scores of IRI and NEO-FFI. EC correlated significantly with FS (r = 0.39, *p* = 0.03; Pearson correlation, two-sided). Furthermore, neuroticism correlated negatively with extraversion (r = − 0.42, p = 0.02) and agreeableness with conscientiousness (r = 0.41, *p* = 0.03). In addition, there were correlations between IRI and NEO-FFI dimensions. PD correlated highly positive with neuroticism (r = 0.78, *p* < 0.001) and negative with extraversion (r = − 0.43, *p* = 0.02). FS correlated positively with extraversion (r = 0.39, *p* = 0.04) and openness (r = 0.40, *p* = 0.03). EC was strongly linked to openness, too (r = 0.51, *p* = 0.005) and to agreeableness (r = 0.44, *p* = 0.02). There were no other significant correlations between personality or IRI dimensions.

**Table 1:** Results of empathy personality questionnaire IRI and personality questionnaire NEO-FFI.

		Females (mean ± standard deviation	Males (mean ± standard deviation)
Empathy Personality Questionnaire IRI	Empathic Concern	16.21 ± 2.20	15.05 ± 2.62
	Personal Distress	10.74 ± 2.70	10.26 ± 2.32
	Perspective Taking	15.94 ± 2.48	14.81 ± 3.11
	Fantasy	14.85 ± 3.04	13.38 ± 2.84
Big Five Personality Questionnaire NEO-FFI	Neuroticism	22.44 ± 8.16	16.20 ± 6.00
	Extraversion	29.64 ± 7.32	28.08 ± 9.12
	Openness	36.48 ± 6.00	37.32 ± 6.00
	Agreeableness	32.88 ± 5.88	28.20 ± 8.16
	Conscientiousness	32.64 ± 7.20	29.28 ± 8.76

As expected, participants found the touch by the real hand of the experimenter more pleasant than the touch by the rubber hand (real hand: 3.31 ± 0.63, rubber hand: 2.11 ± 0.75, t (24) = 5.67, p < 0.001). Furthermore, touch applied by the real hand was also rated to feel stronger than touch received by the rubber hand (real hand: 2.92 ± 0.64, rubber hand: 2.53 ± 0.67, t (24) = 2.99, p < 0.01) (note that for four participants behavioral responses were missing due to technical reasons).

### FMRI results: brain responses to touch

**Figure 1:**
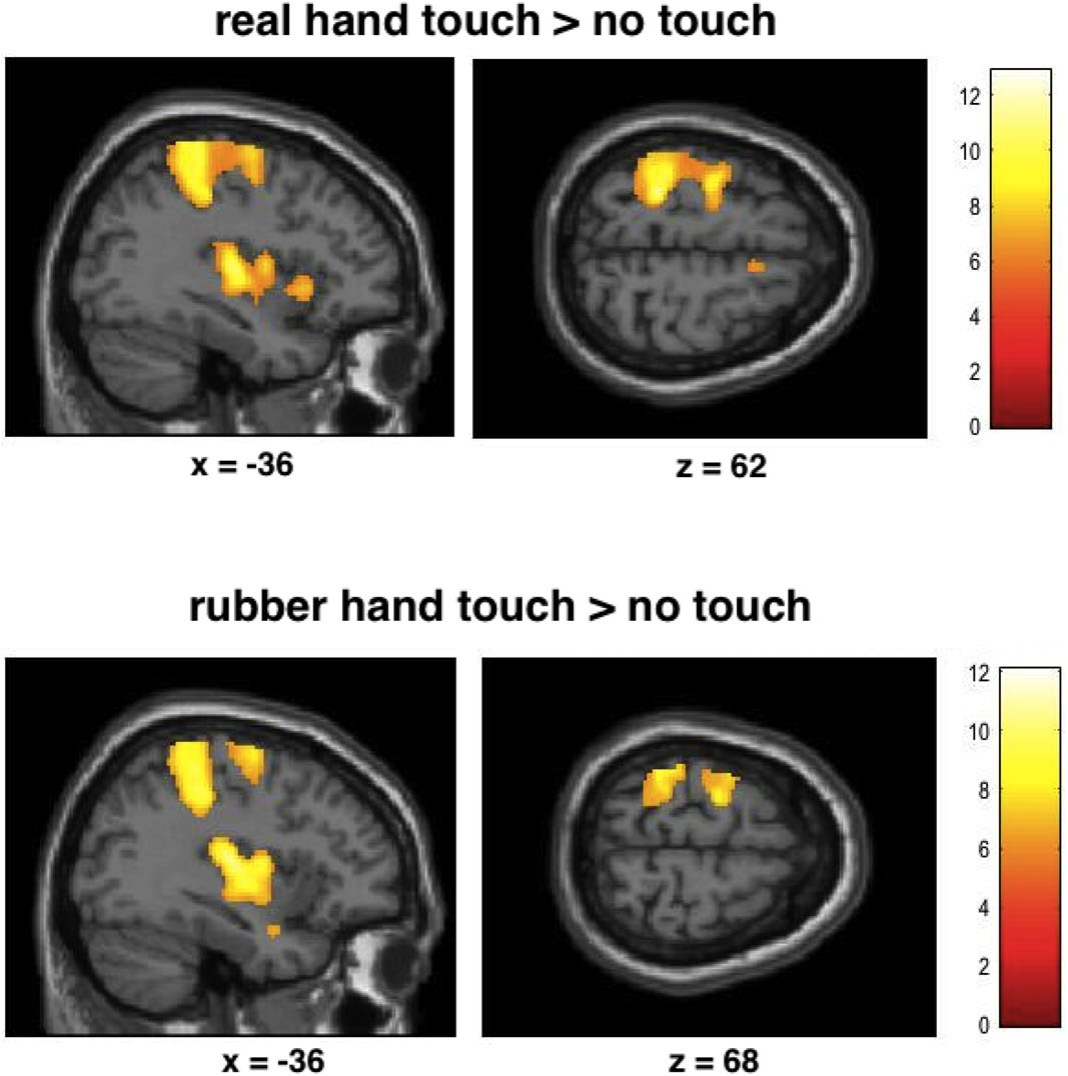
Statistical maps showing brain activation while participants received touch on the palm of the hand by the experimenter’s hand or by a rubber hand (relative to no touch). Areas of significant fMRI signal change are shown as color overlays on the T1-MNI reference brain (FWE corrected at *p* < 0.05).

Brain responses to touch applied by the real hand relative to rest revealed activations in SI, bilateral SII, primary motor cortex, bilateral premotor areas (BA6), inferior frontal cortex, and other brain areas (Fig. 1, *p* < 0.05, FWE corrected). The contrast touch applied by the rubber hand relative to rest revealed similar brain activations (see Table 2).

**Table 2:** Results of random effects analysis for brain responses when receiving touch by a hand and by a rubber hand, respectively (p < 0.05, FWE corrected, L = left hemisphere, R = right hemisphere; in brackets: uncorrected results).

Contrast	Brain region	Peak MNI location (x, y, z)	Peak z-value	Number of voxels
Touch of real hand versus no touch	L SI/	− 26 − 38 64	6.99	6125
	SII/parietal/central operculum	− 54 − 28 20	7.31	
	R middle temporal gyrus	58 − 62 6	6.80	1795
	R SII	62 − 16 20	6.78	
	R posterior insula	42 − 10 4	6.09	849
	R anterior insula	36 24 0	5.81	
	L Thalamus	− 10 − 16 2	5.96	348
	R cerebellum	28 − 52 − 28	5.90	334
	L supplementary motor cortex	− 6 − 6 52	5.36	437
	R SI	40 − 30 46	5.46	106
	R precentral gyrus	38 − 2 54	5.38	35
	Middle cingulate gyrus	− 8 − 20 44	5.62	44
Touch of rubber hand versus no touch	L SI/SII/operculum/insula	− 40 − 22 20	7.09	5258
	R SI/SII	64 − 14 22	7.07	2127
	R middle temporal gyrus	58 − 64 4	6.22	203
	R anterior/posterior insula	42 0 − 10	6.22	424
	L precentral gyrus	− 28 − 12 62	6.20	500
	L Thalamus	− 14 − 24 4	6.17	336
	R cerebellum	28 − 56 − 26	6.05	285
	L middle temporal gyrus	− 54 − 64 10	5.89	271
	R precentral gyrus	36 − 2 52	5.15	20
	L supplementary motor cortex	− 8 6 52	5.14	147
	R amygdala	24 − 4 − 20	5.12	7
Touch of real hand minus no touch vs. rubber hand minus no touch	(R middle /inf. frontal gyrus)	32 22 20	3.80	32
	(L operculum/anterior insula)	− 40 − 10 8	3.52	16
	(L thalamus)	− 20 − 8 12	3.44	13
	(R postcentral gyrus)	34 − 18 30	3.31	5
Touch of rubber hand minus no touch vs. real hand minus no touch	(R/L precentral gyrus)	2 − 28 68	4.35	499
	(L SI/supramarginal gyrus)	− 58 − 28 46	4.18	148
	(L occiptial cortex)	− 50 − 70 − 8	3.77	137
	(R SI)	60 − 16 42	3.81	65
	(L ant./post insula)	− 36 − 8 14	3.22	7
	(L postcentral gyrus)	− 56 0 30	3.16	5

Comparing brain responses related to touch by the real hand with touch by the rubber hand results revealed no differences (*p* < 0.05, FWE corrected, same for opposite contrast). However, uncorrected results revealed an involvement of insula and SI (see Table 2 for details). Given that the intensity of the rubber hand and skin touch was felt differentially by the participants, we repeated the analysis using the perceived intensity of touch as a covariate. Results for skin relative to rubber touch revealed activity in posterior insula, right middle temporal gyrus, hippocampi, brain stem, and cerebellum. Comparing brain responses for rubber touch relative to skin touch revealed no significant activations (all at an uncorrected threshold of *p* < 0.001).

### FMRI results: brain responses to touch and empathy

**Figure 2:**
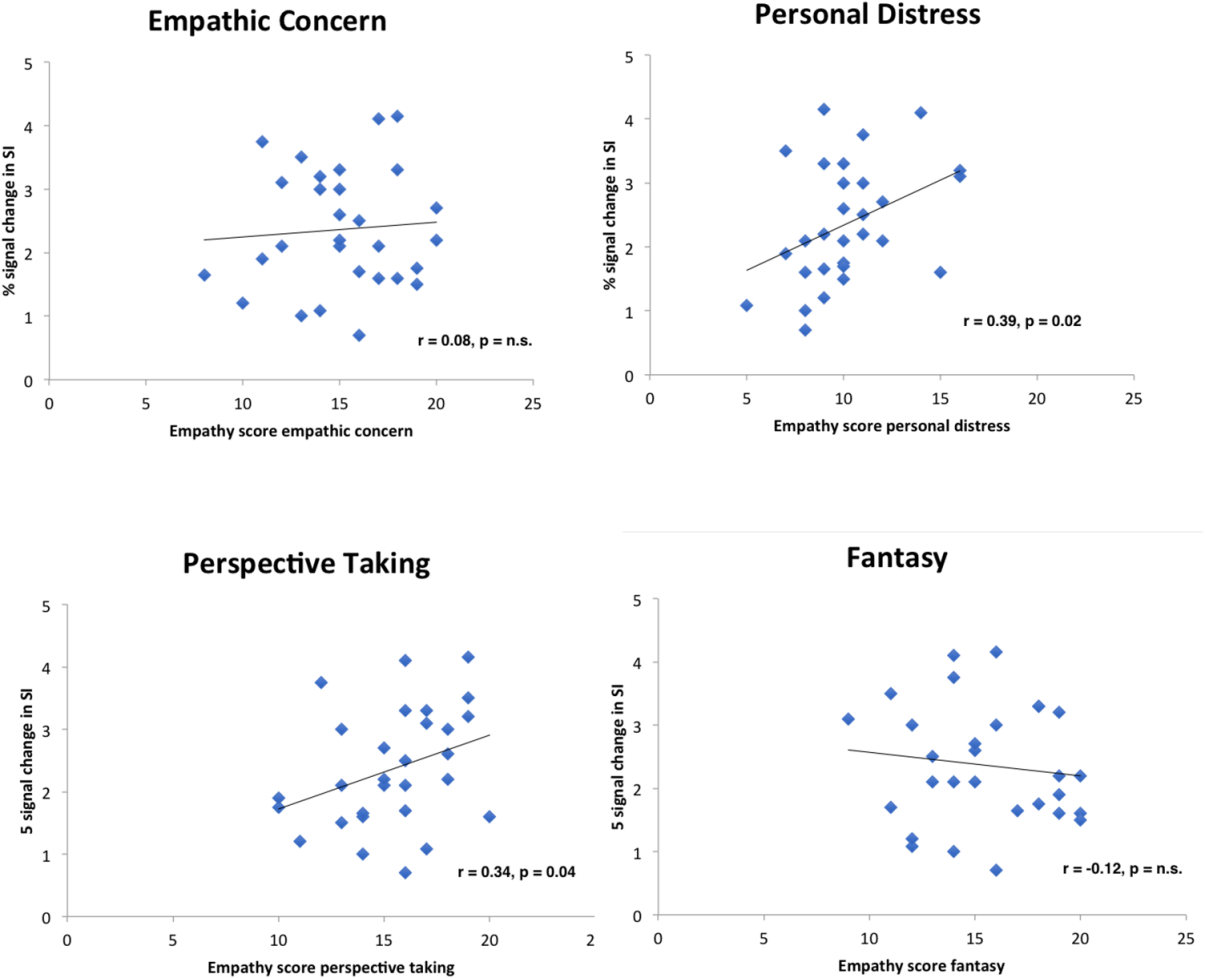
Correlation scatterplots for empathy scores of IRI with BOLD signal change in SI.

Figure 2 displays scatterplots of the relationships between dispositional empathy measures and brain activity in SI. Results revealed that PD and PT correlated significantly positive, whereas FS and EC showed no linear relationship with activity in SI (both for real hand touch and for rubber hand touch) (real hand touch; PD: r = 0.39, *p* = 0.02, PT: r = 0.34, *p* = 0.04, EC: r = 0.08, *p* > 0.10, FS: r = − 0.12, *p* > 0.10; rubber hand touch: PD: r = 0.40, *p* = 0.02, PT: r = 0.32, *p* = 0.04, EC: r = 0.02, *p* > 0.10, FS: r = − 0.21, *p* > 0.10; Pearson correlation). Activity in left SII was not correlated with empathy measures, but right SII displayed positive relationships with PT (real hand touch; PT: r = 0.26, *p* = 0.09; rubber hand touch; PT: r = 0.26, *p* = 0.02; all other dimensions *p* > 0.10).

To test our hypothesis we tested whether dispositional empathy predicts brain activation in SI (real hand touch). We calculated a linear regression analysis in which all four empathy dimensions (EC, PD, PT,FS) and age went simultaneously into one model (R = 0.63, adjR^2^ = 0.27, F(5, 28) = 3.02, *p* = 0.03). Results demonstrated that the empathy score PD was a significant predictor of the brain activation in left SI (real hand touch relative to no touch) (β = 0.36, *p* = 0.04). In addition, PT predicted significantly SI activity (β = 0.40, *p* = 0.03), as well as age (β = 0.38, *p* = 0.03). Other empathy dimensions failed to show significant effects. Analogue calculations for rubber hand touch revealed similar results (see Table 3).

**Table 3:** Regression analyses of brain activity in SI with empathy subscales as predictors.

Contrast	Model					Coefficients (standardized)		
	R	R^2^	Adj. R^2^	ANOVA		Betas	T	Sign
Real hand touch—no touch	0.63	0.40	0.27	F (5,28) = 3.02, *p* = 0.03	EC:	− 0.08	− .41	*p* = 0.69
					PD:	0.36	2.21	***p* = 0.04**
					PT:	0.40	2.29	***p* = 0.03**
					FS:	− 0.02	− 0.12	*p* = 0.90
					Age:	0.38	2.25	***p* = 0.03**
Rubber hand touch—no touch	0.68	0.46	0.34	F (5,28) = 3.92, *p* = 0.01	EC:	− 0.11	− 0.62	*p* = 0.54
					PD:	0.38	2.44	***p* = 0.02**
					PT:	0.39	2.37	***p* = 0.03**
					FS:	− 0.09	− 0.51	*p* = 0.62
					Age:	0.43	2.72	***p* = 0.03**

All four IRI dimensions (EC, F, PT, PD, age) went simultaneously in one model. Significant results in bold.

When adding perceived strength and pleasantness of the touch as predictors the model, results were similar, but the model did not improve. Furthermore, those variables were no significant predictors. In addition, adding gender did not change the results and reduced the fit of the model.

We then ran analogue regression analyses for right and left SII. Results revealed that none of the empathy dimensions predicted brain responses here (no significant models or predictors, *p* > 0.10).

We further tested if somatosensory brain activity in SI and SII were related to the perceived strength or pleasantness of the stimulation. Results revealed no significant results (Pearson correlations, all *p* > 0.10).

**Figure 3:**
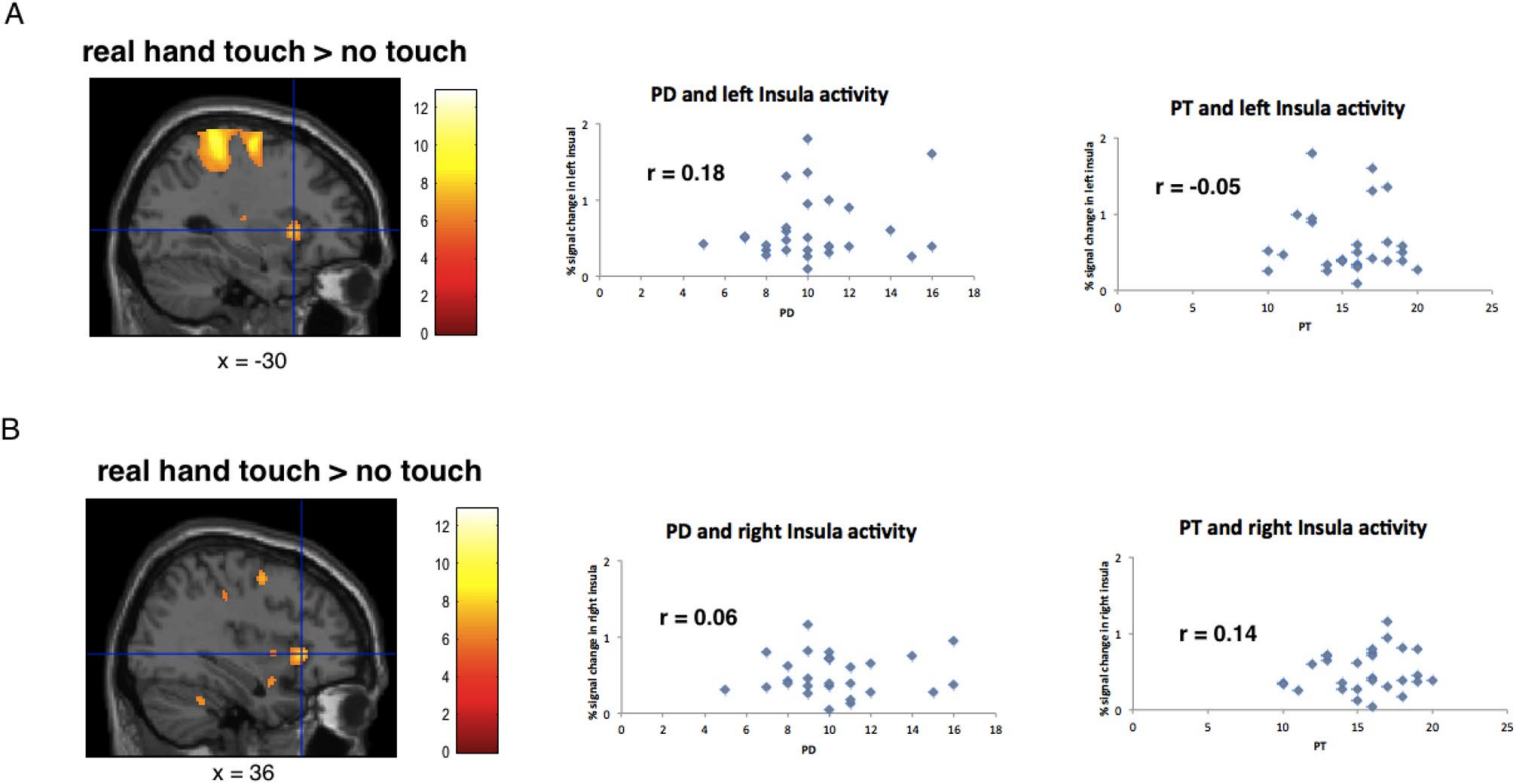
Correlation scatterplots for empathy scores of IRI (PD and PT) with BOLD signal change in left and right anterior insula. Results revealed no significant relationships with any of the IRI dimensions (all *p* > 0.10).

Last, we tested if activity in the anterior insular cortex correlates with empathy dimensions. Pearson correlations revealed no significant results or trends with any of the empathy measures (see Fig. 3, Pearson correlations, all *p* > 0.10).

### FMRI results: brain responses to touch and big five

In order to further assess whether somatosensory brain activation due to felt touch is related to personality dimensions, we tested in an analogue way whether Big Five personality dimensions were associated with brain responses in SI and SII.

Pearson correlations revealed a significant linear relationship for extraversion (negatively) and a trend for neuroticism with SI activity (real hand touch; E: r = − 0.34, *p* = 0.04, N: r = 0.31, *p* = 0.05, other dimensions *p* > 0.10; rubber hand touch: E: r = − 0.30, *p* = 0.05, N: r = 0.32, *p* = 0.05, other dimensions *p* > 0.10).

We then tested whether the NEO-FFI dimensions predict brain activation in SI (real hand touch) by computing a linear regression analysis, in which all five dimensions and age went simultaneously into the model. Results showed no significant results (R = 0.55, adjR^2^ = 0.12, F(6, 28) = 1.63, p > 0.10; all predictors p > 0.10) (similar results for rubber hand touch). Analyses for left and right SII and for insula similarly failed to reveal significant predictors of the Big Five.

## Discussion

The present study aimed to test whether brain responses in somatosensory cortices caused by a touching hand are associated with empathy personality measures. Results revealed that empathy measures PD and PT predicted brain responses in SI both for touch by real hand and touch by a rubber hand.

Based on the present data, it must remain unclear, whether the relationship between empathy and somatosensory activity reflects a top-down or a bottom-up process. A bottom-up view would argue that somatosensory activation would cause higher attention and thereby result in higher empathy scores (e.g.,^28^). For example, Schirmer et al. support this view of a bottom-up somatosensory processing by demonstrating that touch sensitizes ongoing cognitive and emotional processing^40^. A top-down view would explain the results by understanding empathy as a simulation process^1,52,53^. For example, Rizzolatti et al. argue that we understand others through an “internal act, that recaptures the sense of their acting”^54^. In this view, we understand our conspecifics by simulating other’s actions, sensations, or feelings. In the current experiment participants may not observe someone’s action visually, but through their tactile senses. Thus, the results may reflect simulation processes of the toucher’s intention, based on observation via the tactile sense of our participants (even when intentionally applied by a rubber hand). Alternatively, one could also argue that the more accurate we are to our own sensations, the more we are likely to be attuned to other’s bodily sensations. In this way, empathy personality traits may be associated with the strength of brain activation in SI because empathic individuals pay more attention both to other’s and the own sensations. An example for this relationship is a recent study, which demonstrated that meditation (paying attention to the own body) improved tactile processing^38^. Similarly, Bauer et al. reported that sustained attention to the right or left thumb in the absence of any external stimuli activates somatosensory brain areas, suggesting that top-down interoceptive attentional processes can modulate primary somatosensory areas^55^.

What are the underlying neural substrates of our results? Based on the present experiment it is difficult to address this question. Considering our approach of an exploratory correlative study, we cannot identify (or rule out) a single mechanism that may account for the correlations we report. Different processes may explain our findings. For example, measures not related to empathy might have caused the significant correlations (e.g., prosocial personality dispositions not examined in this study). Furthermore, given that we have compared brain activity with an offline-behavior (empathy questionnaire), we also have to stress that the correlation we found must not necessarily mean that the voxels of those brain activity are linked to the underlying concept targeted by this questionnaire.

The somatosensory system engages a widespread cortical network including SI, SII, motor, premotor, inferior parietal regions, opercula and insular regions^56,57^. Recently, a ventral pathway of somatosensory perception, linked to perception and recognition of tactile stimuli (analogue to the visual modality), has been reported. This stream originates from SI, passes SII, and then terminates in the insula^58,59^. One could speculate that the insula, which is known as an interface between cognitive and affective processing, may be the neural substrate of the link between empathy and touch we here report^60,61^. However, the present data did not find insula activity linked with empathy personality measures. Future studies may further test which of these brain areas may underly the relationship between empathy and tactile processing.

The current study demonstrates relationships of somatosensory brain activating with PD and PT, but not with any other dimensions of the IRI. The PT dimension of empathy has been linked to the cognitive part of empathy. Our results confirmed previous studies on mirror-like activations in somatosensory cortices during observation of touch, which showed significant relationships with the PT dimension (e.g.,^35,36^). In contrast to PT, PD addresses an affective form of empathy that describes aversive emotional feelings such as anxiety of fear when witnessing someone else in pain. Several studies linked the PD dimension of empathy with somatosensory activity. For example, Banissy et al. reported grey matter volume differences in somatosensory brain areas (and insula) linked to PD^16^., Ashar et al. used machine learning-based regression approaches and suggested two dissociable brain systems for empathic care and distress, whereas distress was associated with premotor and somatosensory cortical activity (SI and SII)^26^. Furthermore, the mu suppression in sensorimotor cortex, which is a marker for empathy of pain and emotional contagion, was found to be positively correlated with PD^62^. Peled-Avron et al. reported this marker for sensorimotor resonance also to be correlated with PD when participants observed social touch^63^.

Our results also revealed differences in brain activation for real hand touch relative to rubber hand touch (at an uncorrected level). The results are in line with previous studies on skin-to-skin touch relative to indirect touch. For example, Kress et al. showed stronger activation in somatosensory cortices and insula for skin-to-skin touch^64–66^. Numerous studies have shown an important role of the insula in affective touch, but affective touch also seems to engage SI^67,68^.

We also report an effect of age on tactile processing. This is in line with previous studies showing that age is associated with increased somatosensory activity, which may be explained as a compensation for deficits in the ageing brain. However, previous studies typically observe these changes when comparing old (above 60 years) with young (for example 20 years) participants (e.g.,^69^). Here we included predominantly young participants with relatively low standard deviations of age. Future studies are needed to further elaborate whether somatosensory activations even in young participants may show ageing effects.

In this study we also examined the relationship between Big Five dimensions and somatosensory activity. Our results replicated previous results by showing significant negative correlations with extraversion^70,71^. Thus, the more introverted individuals were, the higher was the cortical activity in SI. This finding is in line with the theory of Eysenck, which states a relation between cortical arousal and sensitivity, hypothesizing that “arousal messages” from the ARAS (ascending reticular activating system) and the visceral brain may facilitate the detection of weak stimulation by raising the cortical arousal^72^. While we replicated the link of SI with extraversion, our previous results did not find any relationships with the IRI dimensions. This seems to be remarkable, suggesting that in contrast to touch given by a real experimenter touch provided by a non-human device (as in our previous study) does not seem to affect our empathic brain areas. Moreover, we here demonstrated that touch by a rubber hand reveals similar results. Therefore, touch reaches our empathy brain circuits when it is intentional done by a human, not by a machine. But this touch does not have to be done by a human hand itself, a rubber hand is sufficient if the participant is believing that this hand is conducted by the experimenter (similar^68^).

Several limitations of this study have to be mentioned. First, the number of participants is rather small for studies examining personality traits. Future studies should increase the number of participants. Second, in order to assess gender effects, the variable gender (experimenter and participant) could have been systematically manipulated. Third, when correcting our results for multiple comparisons, results revealed partly only a trend for significance. Thus, further studies are needed to replicate the effects of this pilot study.

Our results suggest that empathy can be separated in different parts and these dimensions may have dissociable underlying neural substrates, including the somatosensory cortices (SI and SII). Thus, the roles of the somatosensory cortices may be more complex than previously thought. Whereas in the traditional view these brain areas represent touch on the body surface in a more or less mechanical way, recent studies suggest that these brain regions may also be important for empathy or emotional regulation in general. For example, it has been shown that sensorimotor regions are related to feelings of guilt^73^. Moreover, many studies report an involvement of the somatosensory cortices on various levels of emotional processing. For example, Kropf et al. suggested that the somatosensory cortices may be involved in interoceptive attention and the generation and regulation of emotional state^74^. This is supported by numerous studies reporting an involvement of the somatosensory cortices in patients with mental disorders associated with abnormal emotional regulation, such as major depression, bipolar disorder, and posttraumatic stress disorder. For example, a study on patients with panic disorder reported an increased functional connectivity between the somatosensory cortices and both the thalamus and left dorsal anterior cingulate cortex^75^. Consequently, it is hypothesized that the somatosensory cortices might be a treatment target for some mental disorders that are particularly linked to emotional deregulation^74^. Recent studies on empathy (or compassion) trainings or meditation provide first encouraging results^38,39^.
